# Moderating or mediating effects of family characteristics on socioeconomic inequalities in child health in high-income countries – a scoping review

**DOI:** 10.1186/s12889-022-12603-4

**Published:** 2022-02-17

**Authors:** Stephanie Hoffmann, Lydia Sander, Benjamin Wachtler, Miriam Blume, Sven Schneider, Max Herke, Claudia R. Pischke, Paula Mayara Matos Fialho, Wiebke Schuettig, Marie Tallarek, Thomas Lampert, Jacob Spallek

**Affiliations:** 1 Department of Public Health, Brandenburg University of Technology Cottbus-Senftenberg, Universitaetsplatz 1, 01968 Senftenberg, Germany; 2 Department of Epidemiology and Health Monitoring, Robert Koch Institute, Berlin, Germany; 3 Center for Preventive Medicine and Digital Health Baden-Württemberg (CPD-BW), Medical Faculty Mannheim, Heidelberg University, Mannheim, Germany; 4 Institute of Medical Sociology, Medical Faculty Martin-Luther-University Halle-Wittenberg, Halle, Germany; 5 Institute of Medical Sociology, Centre for Health and Society, Medical Faculty, Heinrich Heine University Duesseldorf, Duesseldorf, Germany; 6 Chair of Health Economics, Technical University of Munich, Munich, Germany

**Keywords:** Child, Family, Health, Health status disparities, Infant, Socioeconomic factors, Socioeconomic health inequalities

## Abstract

**Background:**

By explaining the development of health inequalities, eco-social theories highlight the importance of social environments that children are embedded in. The most important environment during early childhood is the family, as it profoundly influences children’s health through various characteristics. These include family processes, family structure/size, and living conditions, and are closely linked to the socioeconomic position (SEP) of the family. Although it is known that the SEP contributes to health inequalities in early childhood, the effects of family characteristics on health inequalities remain unclear. The objective of this scoping review is to synthesise existing research on the mediating and moderating effects of family characteristics on socioeconomic health inequalities (HI) during early childhood in high-income countries.

**Methods:**

This review followed the methodology of “Preferred Reporting Items for Systematic reviews and Meta-Analyses extension for Scoping Reviews”. To identify German and English scientific peer-reviewed literature published from January 1^st^, 2000, to December 19^th^, 2019, the following search term blocks were linked with the logical operator “AND”: (1) family structure/size, processes, living conditions, (2) inequalities, disparities, diversities, (3) income, education, occupation, (4) health and (5) young children. The search covered the electronic databases PubMed, PsycINFO, and Scopus.

**Results:**

The search yielded 7,089 records. After title/abstract and full-text screening, only ten peer-reviewed articles were included in the synthesis, which analysed the effects of family characteristics on HI in early childhood. Family processes (i.e., rules /descriptive norms, stress, parental screen time, parent–child conflicts) are identified to have mediating or moderating effects. While families’ living conditions (i.e., TVs in children’s bedrooms) are suggested as mediating factors, family structure/size (i.e., single parenthood, number of children in the household) appear to moderate health inequalities.

**Conclusion:**

Family characteristics contribute to health inequalities in early childhood. The results provide overall support of models of family stress and family investment. However, knowledge gaps remain regarding the role of family health literacy, regarding a wide range of children’s health outcomes (e.g., oral health, inflammation parameters, weight, and height), and the development of health inequalities over the life course starting at birth.

## Background

Extensive evidence demonstrates the existence of health inequalities across the entire course of life [1–5]. Early childhood, however, has been found to be particularly relevant for the development of health behaviour and for health in the long-term [2]. This life stage spans several age groups, including newborns (birth to 1 month), infants (1 month to 1 year), toddlers (1 year to 2 years), and preschoolers (2 years to 6 years) [6]. Health in early childhood depends on the families’ socioeconomic position (SEP), which is commonly operationalised by parental education, occupation and/or income. In general, socioeconomically disadvantaged children show disproportionately poorer health outcomes compared to children from families with a higher SEP. For example, children under the age of six from families with a lower SEP are more likely to have asthma, a delayed cognitive development, a higher prevalence of overweight/obesity, and lower levels of physical activity [7–11].

The circumstances and pathways by which SEP affects children’s health are complex. Over the last two decades, explanatory theories of health inequalities have increasingly considered eco-social perspectives [12, 13]. For example, Krieger’s eco-social theory (2001) posits that the human embodiment of health inequalities results from interactions between biological processes and the social environments humans inhabit [13, 14]. Individual health outcomes, therefore, cannot be understood independently from social environments. In early childhood, the characteristics of the family as the most important surrounding profoundly influences children’s health [15, 16]. For example, marital status, parenting styles and family living conditions are associated with health in this age group [17–19]. Various studies indicate that stressful parental partnerships, incoherent parenting practices, and unfavourable parental health behaviour are more frequently observed among families with lower SEP [20–23]. Following these eco-social perspectives, it can be assumed that the embodiment of health inequalities may result from a dynamic interplay between individuals and families’ characteristics such as processes (e.g., parenting, parental collaborating), structures/size (e.g., marital status), and living conditions.

To seek for explanatory mechanisms, two frequently cited models follow assumptions of social causation [24]. First, the family stress model (FSM) proposes that economic hardship (e.g., low income, job loss) aggravates parents’ psychological distress (e.g., hopelessness, hostility) which leads to inter-parental conflicts. This may, in turn, influence health outcomes in early childhood through inconsistent or harsh parenting practices [24, 25]. Second, the family investment model (FIM) focuses on the economic, educational, and occupational circumstances of a family that determine the resources parents can invest in their children. These investments contribute to children’s development and health in early childhood and later life. The dimensions of parental investment may be as manifold as follows: behaviour (e.g., parent–child time spent, promotion of extracurricular activities), nutrition (e.g., sufficiently nutritious diets), education (e.g., learning materials available at home), health behaviour and health (e.g., utilization of medical care, parental health behaviour, rules restricting of media use), and living conditions (e.g., residence) [25–28]. The FIM and FSM reflect theoretical assumptions of family’s mechanisms underlying health inequalities, which only have been studied with regard to few health conditions among children [29]. However, in line with the assumptions of social causation (e.g., FSM, FIM), family characteristics could depend on the SEP which, in turn, affects children’s health (defining mediating effects). Family characteristics might also affect the association between SEP and children’s health (defining moderating effects) [30].

Although it is known that family SEP determines differences in health outcomes and influences family characteristics, the specific pathways by which family SEP affects health behaviour and health in early childhood in high-income countries remain unclear [31]. Therefore, the objective of this scoping review was to synthesise the extent, the nature, the results, and the knowledge gaps of existing research on families’ characteristics underlying health inequalities by exploring:

1. which family characteristics mediate the association between SEP and health of young children (mediating effects), and
2. which family characteristics moderate the association between SEP and health of young children (moderating effects).

## Methods

### Scope of the search

This scoping review synthesised qualitative and quantitative research on the mediating and moderating effects of family characteristics (context) on health inequalities (concept) in early childhood (population) in high-income countries. The objective was conceptualised by the key elements of population, concept, and context [32]. To assess the scope of the research on the topic of interest, the methodology for this scoping review was based on the checklist outlined by Tricco et al. (2018): “Preferred Reporting Items for Systematic reviews and Meta-Analyses extension for Scoping Reviews” (PRISMA Extension for Scoping Reviews) [33]. The review protocol was published a priori [34].

**Fig. 1:**
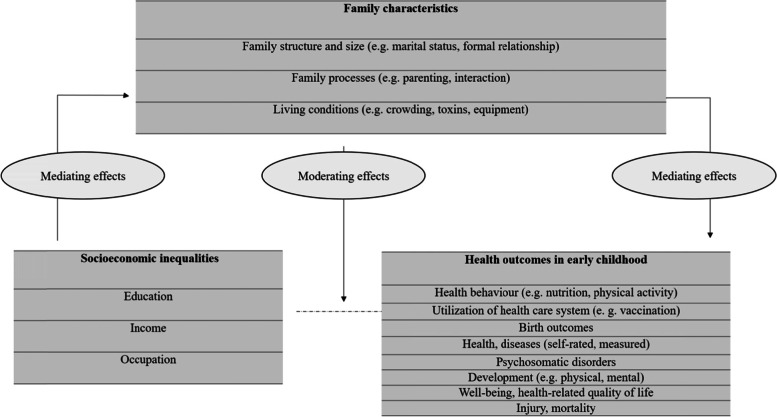
Conceptual Framework of the Scoping Review

Figure 1 illustrates the key elements to conceptualise the research objectives of the scoping review that are based on the Population/Concept/Context framework. The core *concept* pertains to health that depends on the socioeconomic position (SEP) of the family, which is operationalised by parental education, occupation and/or income. The review considered manifold outcomes in health and development in the early childhood (0–6 years, *population*). The *context* encompasses the family as the most important surrounding of young children, which has different structures, processes and conditions.

### Identification in systematic search

As outlined in the review protocol [34], the search strategy was applied to PubMed, PsycINFO (via EBSCO), and Scopus to identify German and English scientific literature published from January 1^st^, 2000, to December 19^th^, 2019 following the PRISMA Extension for Scoping Reviews [33]. The database selection was based on the specific content coverage of peer-reviewed articles within Biomedical and Life Sciences (PubMed), Behavioural Sciences and Mental Health (PsycINFO), and Health Sciences and Education (Scopus).

Search terms were based on key elements of the review objectives that were integrated in the conceptual framework of this scoping review (Fig. 1). Search terms covered free-text words, subject classifications, such as medical subject headings (PubMed) and index terms (PsycINFO) [35]. In order to ensure quality, search terms were refined and checked in consideration of existing guidelines [36].

### The following five search term blocks were designed and linked with the logical operator “AND” to explore the review objective:

- Context: Family characteristics
  - Family structure and size, family processes, family living conditions
- Concept: SEP related inequalities in health outcomes
  - Inequalities, disparities, diversities
  - Socioeconomic position, income, education, occupation
  - Health behaviour, health, development, mortality
- Population: Early childhood
  - Newborn, infant, toddler, preschooler, child

The search strategy was first developed for PubMed and then adapted for use in PsycINFO and Scopus. An outline of the database-specific search strategy was published elsewhere [34].

### Screening and eligibility criteria

The two-step data screening included content screening of titles and abstracts, followed by a full-text screening of the remaining articles. After the elimination of duplicates, two reviewers independently completed the first step (SH, MB), as well as the second step (SH, LS). In the case of contradictory decisions, agreement was reached via discussion between the two reviewers or consultation with a third researcher (JS) with sufficient expertise. To assess inter-observer bias, generalised kappa-type statistics [37] were calculated for both title/abstract screening and full-text screening. To ensure that the reviewers interpreted eligibility criteria similarly, 100 abstracts were randomly selected and jointly discussed.

Peer-reviewed journal articles were included, if they reported on key dimensions of the review objectives. Studies were included, if they described family characteristics (context), such as family structure/size, family processes, family living conditions or parental characteristics (e.g., health literacy) [﻿^19^, ^23^, ^38^–^40^]. To identify health inequalities (concept), all articles had to consider differences in families’ education, income or occupation [41] that influence health outcomes. These were operationalised by health behaviour, utilisation of the healthcare system, health, etiology of communicable and non-communicable diseases, birth outcomes (e.g., birthweight), development, well-being, or mortality in early childhood (0 to 6 years) (population). Furthermore, studies were only included if they had been conducted in high-income countries as defined by the United Nations [31], and published between 01.01.2000 and 19.12.2019 in German or English. According to the scoping review methodology [33], studies with various designs (e.g., cross-sectional studies, longitudinal studies, qualitative studies, and reviews) were eligible for inclusion. Articles were included, if data analyses strived for exploring family characteristics underlying early health inequalities (e.g., moderator analyses, mediator analyses, qualitative analyses [30, 42]. Data analyses were also sufficient for inclusion, if they were reported descriptively (e.g., in reviews) or were represented exclusively in figures or graphs.

Articles that exclusively focused on i) older population groups (i.e., 6–18 years olds) or ii) other contexts than families (e.g., homeless children, children in foster care, institutionalised living, neighbourhood characteristics and environmental exposures) were excluded from the literature synthesis. The exclusion criteria were published a priori [34].

### Data charting and data items

A data-charting form was jointly developed and independently filled in by two reviewers (SH, LS). Data from all articles/studies were extracted doubly, and inconsistencies were resolved in discussion between the two reviewers or via consultation with a third researcher (JS).

For studies that met all the eligibility criteria, data items were summarised in tabular form in accordance with the key dimensions of the review: family characteristics, inequalities in health, children´s age during outcome collection, and an open field for population characteristics. Additionally, data about the country, data collection and data analysis methods, and the main results were documented.

### Synthesis of results

The findings were narratively synthesised following the PRISMA Extension for Scoping Reviews [33]. For illustrative purposes, the findings were mapped using the conceptual framework of this scoping review in Figs. 3, 4, and 5.

## Results

### a. Selection of sources of evidence

**Fig. 2:**
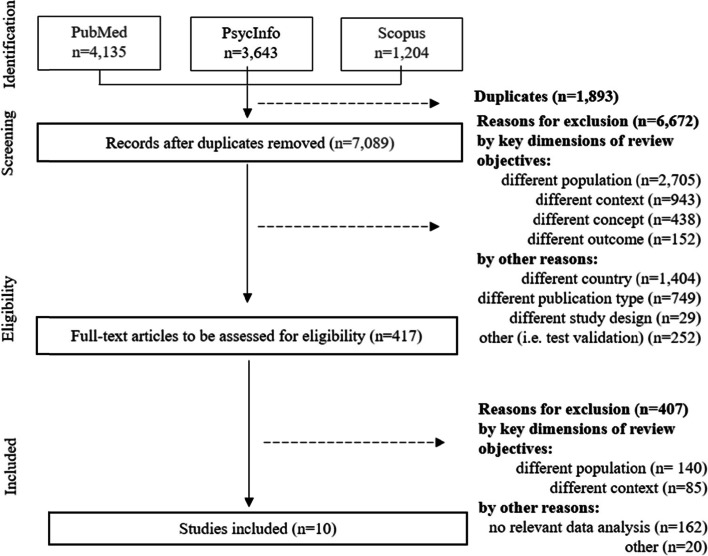
Flowchart of Study Selection Following PRISMA Extension for Scoping Reviews

After removing duplicates, 7,089 articles were included in the screening process. Based on the title and abstracts, found articles were excluded despite matching search terms, because the terms used in the search algorithm corresponded with i) a different population (i.e., children older than 6 years), ii) contexts other than families, and iii) different concepts (i.e., spatial socioeconomic deprivation). Ensuing title/abstract screening, 417 full texts were retrieved and controlled for eligibility. A total of ten peer-reviewed studies met all inclusion criteria. Observer agreement was categorised as excellent in each data selection process (title and abstract screening: Cohen’s Kappa = 0.89; full text screening: Cohen’s Kappa = 0.92). Fig. 2 outlines the selection process.

Figure 2 details database-specific results of the literature search and the numbers of articles screened at each stage in the two-step screening processes as well as reasons for exclusion at both the title/abstract-level (*n* = 6,672) and the full-text level (*n* = 407).

### b. Characteristics of sources of evidence

The majority of the research included in the screening (*n* = 7,089) examined the impact of family characteristics on health, regardless of the SEP or vice versa. Thus, peer-reviewed articles published on socioeconomic health inequalities among young people during the past two decades did rarely consider family characteristics as mediating or moderating factors (*n* = 10).

The studies applied single indicators of income and education [43–49], as well as combined indices thereof [50–52]. No study considered occupation as a dimension of SEP. While one study did not report the methods of data collection [52], all other studies included self-reported information of SEP and health outcomes (*n* = 9). Only a few different outcomes were used, namely screen time (*n* = 2), behavioural difficulties (*n* = 2), development (*n* = 2), parent-rated health (*n* = 2), injuries (*n* = 1) and being breastfed (*n* = 1). While all outcomes in children’s development were measured [49, 52], all other outcomes were reported either by both parents (*n* = 7) or by the mother only [44].

Family characteristics were operationalised by four domains:

1. processes [45–47, 49–52],
2. structure/size [43, 48, 49],
3. living conditions [44, 47], and
4. a combination thereof [49].

With the exception of parental screen time, the studies did not operationalise parental health literacy and did not consider intergenerational relations. With regard to data collection, one study applied observations and video recording [52], while the other studies were based on self-reports on family characteristics (*n* = 9). Although some of the studies drew their sample from a larger longitudinal study [44, 47, 48, 52], the family characteristics data of all the studies were examined cross-sectionally.

The findings mainly referred to children at the age of nine months to six years (sample sizes: 68–14,378), only one study considered newborns [43].

In terms of the geographic scope, 50% of the studies were conducted in the United States of America (*n* = 5). Other countries were the United Kingdom (*n* = 1), Canada (*n* = 1), Finland (*n* = 1), the Netherlands (*n* = 1), and Australia (*n* = 1). There was a notable absence of scientific literature from high-income European and Asian countries [31]. All the studies were published after 2010.

**Fig. 3:**
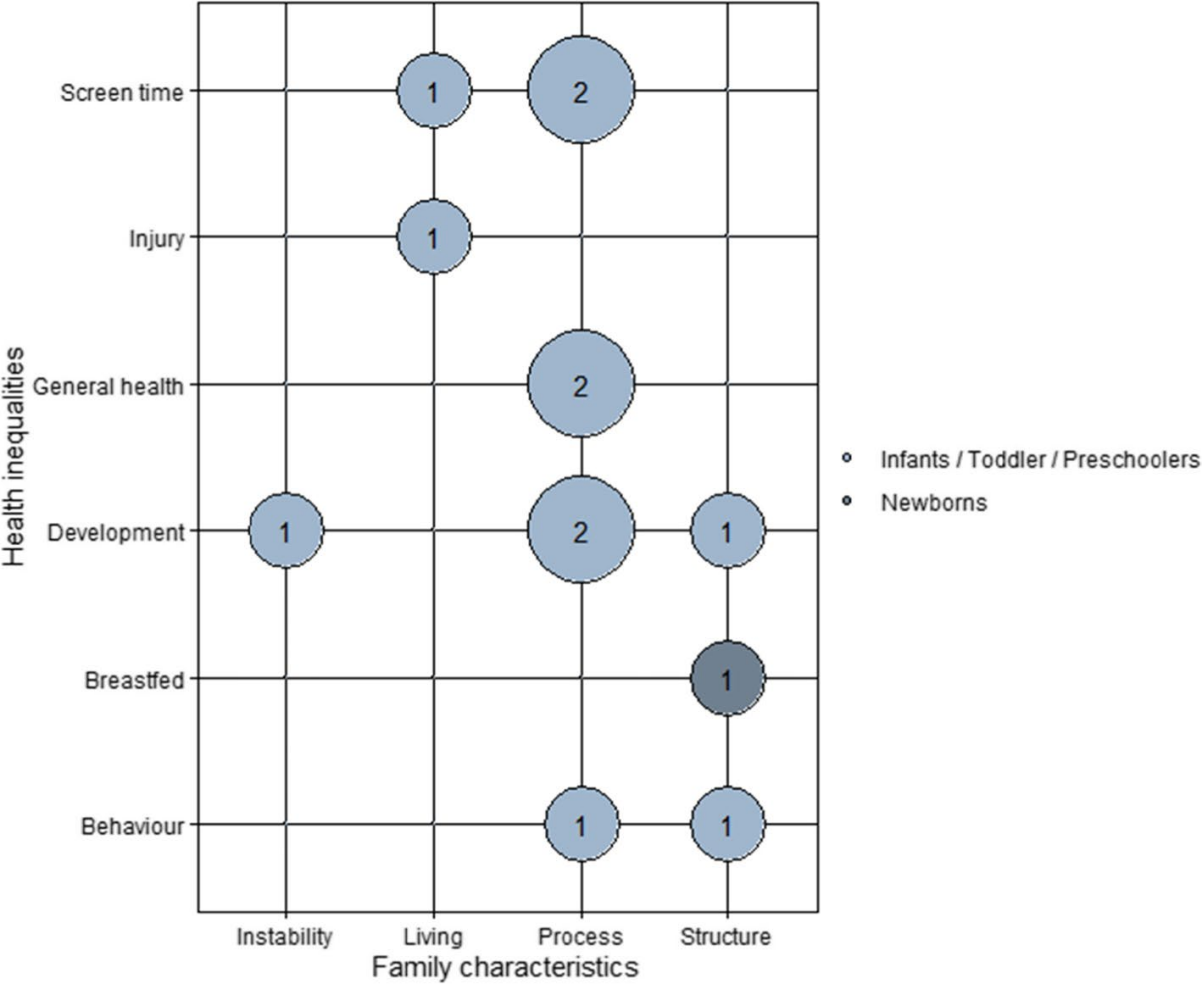
Overview of Studies Examining the Pathways Underlying Health Inequalities with Family Characteristics

Figure 3 provides an overview of the number of studies that examined family characteristics and socioeconomic inequalities in health among young children (i.e., newborns, infants/toddler/preschoolers).

### c. Results of individual sources of evidence

Table 1 provides a detailed description of the studies included and data analyses conducted on mediating or moderating effects of family characteristics.

**Table 1:** Final Individual Sources of Evidence Included in the Scoping Review

Author, Year	Country	Age of Children	Study Size	Other Population Characteristics	Family Characteristics	Family Socioeconomic Position	Health Outcome	Data Analyses
Kim & Gallien 2016 [43]	United States	0–6 months	13,138	no information reported	structure (single-/two-parent: dichotomous)	household income: low/high	being breastfed: dichotomous	moderating effects logistic regression SEP-stratified outcome measures
Pearce et al. 2012 [44]	United Kingdom	9 months –3 years	14,378	excluded: unemployed mothers	living (safety equipment use: score)	maternal education: 7 categories	unintentional injury: dichotomous	mediating effects logistic regression mediating effects Poisson regression
Browne & Jenkins 2012 [45]	Canada	20 months –6 years	501	oversampled: immigrant background	process (differential negativity in family: score)	maternal education: number of years	parent-rated health: 5-point Likert scale	moderating effects logistic regression interaction terms SEP*family processes graphical analysis/chart
Puff & Renk 2014 [50]	United States	2–6 years	119	no information reported	process (parent–child stress: score)	SEP index (financial cutbacks): score SEP index (negative economic events): score	behavioural difficulties: score	mediating effects linear regression
Määttä et al. 2017 [46]	Finland	3–6 years	864	no information reported	process (rules / norms limiting screentime in hours, minutes: continuous) processes (parental screentime hours per day: metric)	parental education: low/middle/high	screentime: calculated daily average	mediating effects linear regression
Wijtzes et al. 2012 [47]	The Netherlands	4 years	2,786	excluded: non Dutch mothers	process (parental screentime: scale) living (TV in bedroom: dichotomous)	maternal education: 4 categories	screentime: dichotomous	mediating effects logistic regression
Strazdins et al. 2010 [48]	Australia	4–5 years	3,580	no information reported	size (infant in family: dichotomous) structure (single-/cohabiting: dichotomous)	household income: low/mid-to-high	behavioural difficulties: score	moderating effects linear regression, chart SEP-stratified outcome measures
Hagan et al. 2016 [51]	United States	4–6 years	338	no information reported	processes (negativity in parent–child relationship: score)	SEP index (education, income): low/high	parent-rated impairment: score parent-rated chronic medical conditions: score	mediating effects reported moderating effects linear regression interaction terms SEP*family process graphical analysis/chart
Li et al. 2017 [49]	United States	5–6 years	140	included: uncomplicated, singleton pregnancies	size (children in household: number) size/living combined (household instability: score) process (major life events: scale)	family income: 10 categories	development in cognitive self-regulation: score	mediating effects linear regression moderating effects linear regression interaction terms family size*SEP
Demir et al. 2015 [52]	United States	5–6 years	68	included: children with brain injury, English-speaker	process (parent–child talk: metric)	SEP index (income, education): Metric	development in language: score	mediating effects linear regression

### d. Synthesis of results

#### Mediating Effects (objective 1) of family processes and family living conditions

In total, seven studies conducted analyses to identify whether family characteristics have a mediating effect on health inequalities. Mediating effects were explored, if SEP was associated with family characteristics, which, in turn, affected health outcomes. To examine those effects, studies presented quantitative data analyses or only reported their findings.

**Fig. 4:**
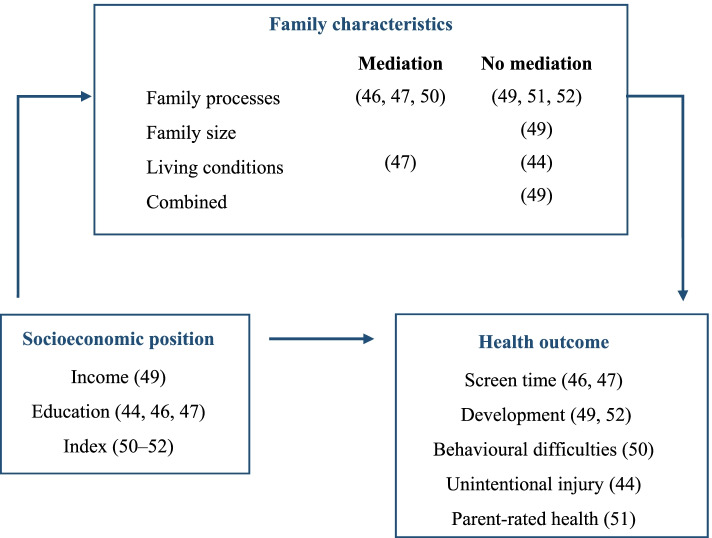
Mediating Effects of Family Characteristics in SEP-Related Health Inequalities in Early Childhood (Objective 1) [46, 47, 50], [49, 51, 52], [49], [47], [44], [49], [49], [44, 46, 47], [50–52], [46 47], [49, 52], [50], [44], [51]

The findings of the studies indicated that family processes mediate the association between family SEP and health among young people regarding the following three domains [46, 47, 50]: 1. parent-rated stress in parent–child relationship, 2. parental rules/descriptive norms, and 3. parental own health-related behaviour. Fewer economic resources were linked to higher parental stress that worsened behavioural difficulties (e.g., uncommunicative, depressed, anxious, attention problems, aggressive behaviours) [50]. Furthermore, a lower parental education was associated with fewer rules/ descriptive norms, resulting in higher daily averages in preschool children’s screen time [46]. Additionally, the parental extent of screen time [46, 47] contributed to the association of low family education and long screen time of children. In terms of family living conditions, a television set in children’s bedrooms conveyed the association between a lower educated maternal background and an extensive screen time among children [47]. Fig. 4 provides an overview of reported mediating effects and illustrates whether effects were found or not. Fig. 4 maps the findings using the conceptual framework of the scoping review by means of presenting each study in which mediating effects were examined, along with their references. The figure illustrates parameters used to operationalise socioeconomic inequalities in health in early childhood as well as the family characteristics considered.

#### Moderating effects (objective 2) of family processes and family structure and size

In total, five studies conducted analyses to identify whether family characteristics have a moderating effect on health inequalities. Moderating effects (*P*-value < 0.05) were statistically tested with interaction terms between SEP and family characteristics using logistic or linear regressions [45, 49, 51], SEP-stratified outcome measures also using either logistic or linear regression [43, 48], or graphical analyses [45, 51].

**Fig. 5:**
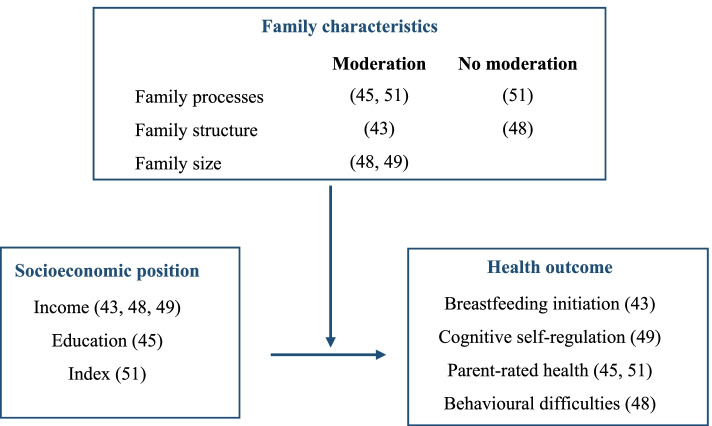
Moderating Effects of Family Characteristics on Health Inequalities in Early Childhood (Objective 2) [45, 51], [51], [43], [48], [48, 49], [43, 48, 49], [45], [51], [43], [49], [45, 51], [48]

The findings of the studies indicate that family processes regarding sibling differences in parenting (i.e., differential negativity) or conflicts in parent–child relations (i.e., negativity) moderate the association between family SEP and children’s health. Children with a lower SEP (i.e., index, education) were more likely to have impairment or poor health. The effect of high negativity within families was shown to be stronger among families with a lower SEP compared to those with high SEP [45, 51]. Moreover, studies suggested moderating effects of both the structure of the family (i.e., single versus married) [43] and the number of children in the household [48, 49]. For example, the association between being breastfed and low-income households was reinforced by single-parent family structure [43]. Furthermore, among low-income compared to high-income families, a greater number of children in the household contributed to an aggravated self-regulation among preschoolers [49]. The moderating effect of having an infant in the family on the behavioural difficulties of preschool children was also shown to be stronger among children from low-income families compared to those from high-income families [48]. Fig. 5 presents all findings and the identified moderating effects.

Figure 5 maps the findings using the conceptual framework of the scoping review by means of presenting each study in which moderating effects were examined, along with their references. The figure illustrates parameters used to operationalise socioeconomic inequalities in health in early childhood as well as the family characteristics considered.

## Discussion

This scoping review provides the first synthesis of scientific peer-reviewed literature addressing the extent, nature, results, and knowledge gaps concerning the impact of family characteristics on health inequalities in early childhood.

Overall, it is notable that only ten studies could be included in the review. Nevertheless, the review reveals specific mediating effects of family processes (e.g., rules/ descriptive norms, stress, and parental screen time) and families’ living conditions (e.g., TVs in bedrooms). Furthermore, negativity in families, single parenthood, and the number of children or infants living in the household emerge as moderators of health inequalities in early childhood.

### Discussion of mediating effects of family characteristics (objective 1)

Parenting is the most frequently investigated family characteristic through which SEP influences preschoolers’ health. *Stress in parent–child relationship *[50], *rules/descriptive norms *[46], and *parental screen time *[46, 47] were found to be family processes with a mediating effect. These findings are in line with both the FSM and the FIM, as they follow the logic of social causation by assuming that differences in SEP lead to differences in parental stress and parental behaviour, resulting in health inequalities, specifically in behavioural difficulties [50] and in screen time [46, 47] among young children. Likewise, existing research examining the (isolated) impact of family characteristics or SEP on health correspond with the findings of this review. There, risk factors for behaviour problems among preschoolers or correlates of sedentary behaviour and screen-viewing among three to seven years old children are associated with both family characteristics and SEP [53–55]. Simultaneously, the systematic literature review on intervention strategies by Altenburg et al. (2016) [56] highlighted the potential impact of parental role modelling (e.g., parental own screen time) on children’s sedentary time. For instance, child-rated parental TV viewing time was found to correlate with screen time in later childhood (eighth grade) [57].

The living conditions of the family, particularly TVs in children´s bedrooms, affected socioeconomic differences in children´s TV viewing time [47]. This result corresponds with evidence on the contribution of living conditions to health-related behaviour among preschoolers. For example, health-related sleeping habits are affected by TVs in bedrooms [58]. Studies on screen time in later childhood and adolescence suggest that the number and placement of TVs are mediators of differences along parental education among adolescents [57].

### Discussion of moderating effects of family characteristics (objective 2)

The studies show that differential negativity in the family (i.e., amount of negativity a child experiences relative to the others) [45] and hostile parent–child relations [51] influence the effects of lower SEP on parent-rated impairment and parent-rated general health. This finding is supported by previous research by Amato et al. (2010) [39], in which stable relationships with an adult caregiver and good parenting were associated with a better health in early life.

The presence of several children in a household was found to moderate the association of low income on lower cognitive competencies [49]. Li et al. (2017) [49] argue that children’s self-regulatory skills do not benefit from a higher number of children (i.e., opportunities for social interaction) in low-income families compared to higher-income families due to related demands on families’ resources. Similarly, Strazdins et al. (2010) [48] conclude that lower family income is associated with behavioural difficulties among preschool children, which is affected by the number of infants in the family. The extent of inequalities in cognitive and behavioural difficulties depend on family structure/size. Thereby, family characteristics contribute to inequalities in health and development in early childhood as they may intensify precarious socioeconomic circumstances.

According to the Kim and Gallien (2016), income-based disparities in breastfeeding initiation depend on single parent status [43]. Empirical literature indicates that an imbalance between work and family life affects breastfeeding initiation and duration, in particular among single mothers [59, 60]. One such study [49] indicated that low family income has an influence on development in early childhood and is affected by single parent status. This result corresponds to existing evidence gained by research on divorce. Amato et al. (2010) [39] and Fincham et al. (2010) [61] showed that marital status contributes to health.

### Remaining research gaps

The effects of SEP on health considering family characteristics may not become apparent during the first years of life compared to higher age groups, because cumulative processes and effects on health throughout the course of life can be assumed [62]. This may be one potential explanation for the small number of studies identified with a special focus on the first months of life [43]. It may also be possible that previous studies applied a rather downstream approach by focusing on individual health and individual level determinants of health only, neglecting the possible impact of social environments and broader societal factors.

The extent of studies on the topic at hand is insufficient to comprehensively assess effects on health inequalities for different reasons. First, the identified literature is mainly descriptive in nature. Second, the findings are limited to a small number of considered i) family characteristics and ii) health outcomes. For example, the studies focus on conservative family structures/sizes (e.g., heteronormative parental relationships, biological parent–child relation, nuclear families). Consequently, the review unveils a number of research gaps that require further investigation in the future. Specifically, multiple levels of family transitions (e.g., parental divorce, re-partnering and remarriage, new half-siblings, and step-families), as well as interrelationships between different generations, should be taken into account [63, 64]. Additionally, this scoping review presents little research on family processes related to parental health literacy. With regard to parental role models, it would be beneficial to examine the mediating or moderating effects of parental health behaviour on the relationship between SEP and child health. Recent studies demonstrate the importance of parental role models in nutritional behaviour and physical activity that influences children’s health [26, 65, 66]. The literature also insufficiently reports on health behaviour (e.g., nutrition, physical activity), as well as health (e.g., child weight, child height, birthweight, oral/dental health) [63]. Third, the studies predominantly refer to educational disparities or families’ income differences. Perspectives on parental employment, however, may also be considered in future research on social gradients in children’s health behaviour and health [67].

### Strengths and limitations

The strength of this scoping review is that it synthesised the international scientific literature on mediating and moderating effects of family characteristics on inequalities in health in early childhood in high-income countries, which, to our knowledge, has not been done before.

One limitation is that despite a large number of children attending day care facilities, this review focuses exclusively on the mediating and moderating effects of the family as the most important social environment in early childhood. The findings presented here should, therefore, be supplemented by reviews of cooperating research groups [68] on the effects of further relevant social environments, such as kindergartens [69] and schools [70], and by reviews examining late childhood and adolescence [29].

Although we have shown that families are an influential environment for young children, individual health and health behaviour are undeniably affected by factors beyond the individual and family level [5, 71, 72] often referred to as “upstream factors” or social determinants of health. These may refer, for instance, to aspects of economic stability, health care, or transport, and are often shaped by policies and contextual factors. Due to the focus of this review on family characteristics, it was not possible to study the impact of upstream factors. Furthermore, this scoping review exclusively considers individual socioeconomic differences in early health in high-income countries as defined by basic economic country conditions (e.g., gross domestic product per capita (GDP)) [31]. Therefore, the results on the mediating or moderating effects of family characteristics are restricted to higher income countries and to young individuals. According to the OECD Social Policy Division [73], there are country-specific differences in family indicators. For example, family size or household size averages vary by country. Further research should take political contexts, such as family politics and labour market policies, into account in order to understand health outcomes beyond the dimension of GDP [74].

This research reviewed correlative associations rather than causal pathways, because family characteristics were examined cross-sectionally by considering one generation of caregivers/parents only. It should be noted that recent literature on family characteristics and children’s health emphasised the impact of family transitions as described above, both in the early course of life [39, 61] and with regard to intergenerational transmission of, for example, parenting styles [75].

Due to the chosen scoping review methodology [33], various sources of research methods were eligible for inclusion. A critical appraisal (i.e., risk of bias) of the studies included was not intended [76, 77]. Rather, the scoping review resulted in a synthesis of peer-reviewed articles of the topic of interest in the last two decades.

The screening and selection process led to difficulties in the generalization of results due to a limited number of studies included. As the review is limited to peer-reviewed articles, it is possible that relevant research published in other formats, such as books, theses, and grey literature, may have remained undetected. The same is true for studies from high-income countries (e.g., France, Italy) that might have been published in other languages than English or German.

## Conclusion

Families are vital social environments with regard to enabling and promoting children’s healthy development. The synthesized research provides a better understanding of the contribution of family characteristics to the association between families’ SEP and health inequalities in early childhood in higher income countries. The studies included were not enough in amount or extent to comprehensively assess the moderating and mediating effect of family processes, family structure/size and living conditions. The small body of evidence identified supports eco-social perspectives on the interacting mechanism between contextual circumstances and individual health. Thus, public health measures for reducing HI and promoting childrens’ health might be more effective when taking children’s family situation, including their resources (e.g., human capital) and environment (e.g., area deprivation), into account.

Future research in public health should (a) validate measures of family characteristics in relation to relevant indicators of children’s health inequalities in early life (e.g., obesity, inflammation parameters), (b) analyse the impact of the family environment on newborns’ and infants’ health inequalities, and (c) clarify causal pathways and mechanisms, for instance through considering parental perspectives (e.g., on practical nexuses, relevant contextual factors and needs). This may be done by combining dimensions from the FIM and the FSM.

## Data Availability

Data sharing is not applicable to this article as no datasets were generated or analysed during the study.

## Abbreviations

SEP: Socioeconomic Position
FSM: Family Stress Model
FIM: Family Investment Model
PRISMA: Preferred Reporting Items for Systematic reviews and Meta-Analyses
